# Intervention dose estimation in health promotion programmes: a framework and a tool. Application to the diet and physical activity promotion PRALIMAP trial

**DOI:** 10.1186/1471-2288-12-146

**Published:** 2012-09-19

**Authors:** Karine Legrand, Emilie Bonsergent, Clotilde Latarche, Fabienne Empereur, Jean François Collin, Edith Lecomte, Evelyne Aptel, Nathalie Thilly, Serge Briançon

**Affiliations:** 1 INSERM, CIC-EC CIE6, Nancy, France; 2 CHU Nancy, Epidémiologie et Evaluation Cliniques, Nancy, France; 3 Université de Lorraine, Université Paris Descartes, EA 4360 Apemac, Nancy, France; 4 Université de Lorraine, Faculté de Médecine, Ecole de Santé Publique, Nancy, France; 5 National conservatory of arts and crafts (CNAM), Nancy, France; 6 Local school office of the Nancy-Metz academy, Nancy, France; 7 Clinical epidemiology and evaluation department, University Hospital of Nancy, Allée du morvan, 54505, Vandoeuvre-lès-Nancy Cedex, France

**Keywords:** Health promotion, Programme, Implementation, Dose, Process, Evaluation

## Abstract

**Background:**

Although the outcomes of health promotion and prevention programmes may depend on the level of intervention, studies and trials often fail to take it into account. The objective of this work was to develop a framework within which to consider the implementation of interventions, and to propose a tool with which to measure the quantity and the quality of activities, whether planned or not, relevant to the intervention under investigation. The framework and the tool were applied to data from the diet and physical activity promotion PRALIMAP trial.

**Methods:**

A framework allowing for calculation of an intervention dose in any health promotion programme was developed. A literature reviews revealed several relevant concepts that were considered in greater detail by a multidisciplinary working group. A method was devised with which to calculate the dose of intervention planned and that is actually received (programme-driven activities dose), as well as the amount of non-planned intervention (non-programme-driven activities dose).

**Results:**

Indicators cover the roles of all those involved (supervisors, anchor personnel as receivers and providers, targets), in each intervention-related groups (IRG: basic setting in which a given intervention is planned by the programme and may differ in implementation level) and for every intervention period. All indicators are described according to two domains (delivery, participation) in two declensions (quantity and quality). Application to PRALIMAP data revealed important inter- and intra-IRG variability in intervention dose.

**Conclusions:**

A literature analysis shows that the terminology in this area is not yet consolidated and that research is ongoing. The present work provides a methodological framework by specifying concepts, by defining new constructs and by developing multiple information synthesis methods which must be introduced from the programme's conception. Application to PRALIMAP underlined the feasibility of measuring the implementation level. The framework and the tool can be used in any complex programme evaluation. The intervention doses obtained could be particularly useful in comparative trials.

**Trial registration:**

PRALIMAP is registered at ClinicalTrials.gov under NCT00814554

## Background

As emphasised by Dusenbury et al. [1] in their review of the implementation of drug abuse prevention in school settings, important variations in the implementation of interventions may arise in health promotion programmes. Other authors before and after him, more particularly Dane and Schneider [2] and Durlak et al. [3] emphasised variation factors, such as those regarding adherence. Nevertheless, the outcomes of programmes are often analysed without taking variability of implementation into account [4]. This can lead to the conclusion that a programme is ineffective when it has not actually been implemented as expected ("type III error " according to Basch et al. [5]). In health promotion programmes, particularly those conducted within the framework of controlled trials, it is therefore necessary to take into account the level to which interventions are implemented when interpreting outcomes. This can be viewed as the dose of intervention received by the target group. The intervention dose must take into account not only the activities performed according to the programme's frame of reference, but also those that were conducted but not planned [6]. This is particularly relevant in health promotion programmes concerning topics for which media coverage may lead to initiatives that are locally driven and independent from the planned programme. For example, nutrition has been the subject of a national programme in France since 2001 [7].

The objective of the present work was to build a framework and to propose a tool with which to measure the quantity and the quality of health promotion activities implemented, whether planned or not, related to the themes of the intervention under investigation. The framework and the tool used to assess the intervention dose, were applied to data from the PRALIMAP trial (PRomotion de l'ALIMentation et de l'Activité Physique, Additional file 1:Box) [8].

## Methods

### Development of the framework

A working group was set up. The group (comprising the authors of the present paper) included specialists in prevention, health promotion and health evaluation.

A literature review also revealed several relevant concepts that were considered in greater detail by the working group. Various methods have been proposed with which to evaluate the process of health promotion programmes [1,9-17].

### Development of the tool

Such programmes are generally implemented in settings constituting homogeneous intervention groups, during defined period(s) of intervention. We designate them “intervention-related groups” (IRG). An IRG is a basic setting (class, school, hospital, district …) in which a given intervention (education, screening …) is planned as part of the programme and in which programme actors may have particular practices likely to introduce variations in the implementation of the activities planned within the programme's frame of reference and/or in the performance of unplanned activities (beneficial or harmful in ways relevant to the programme). For every IRG and every period of intervention, the process evaluation concerned two major domains: the delivery of the intervention and the participation of those involved, each of which was defined in terms of quantity and quality. Four key questions are to be answered: how much did providers do? Did providers do well? Did targets participate? And did targets participate well?

Three categories of programme actors able to influence implementation of health promotion programmes were identified [2]: supervisors, personnel anchors and targets. Supervisors provide personnel anchors with what they need to carry out the intervention, and oversee its implementation. Anchors have two roles: as receivers of training in the intervention by supervisors, and as providers of the intervention to targets.

**Figure 1:**
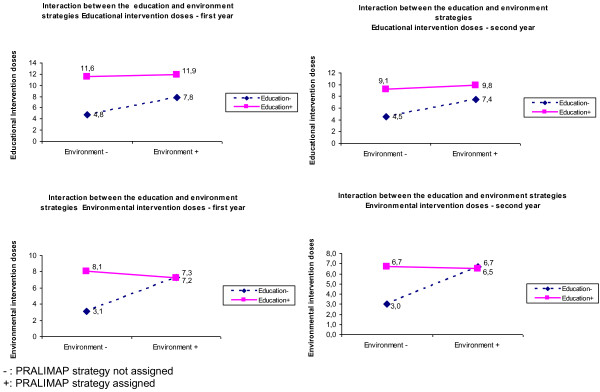
Hierarchical organisation of the 16 key objects of evaluation contributing to intervention dose calculation.

Crossing of both domains (delivery, participation) with both declensions (quantity, quality) gives four levels to be estimated for each type of programme actors (supervisor, personnel anchor (receiver and provider), target), i.e. 16 evaluation objects (Figure 1). In practice, only 12 of the 16 are eligible for the process evaluation because targets do not perform interventions and supervisors do not work in the field. So, indicators are established for every IRG in every period and bracketed in indicator report sheets.

### Testing the tool

The framework and the tool used to assess the intervention dose, were applied to the PRALIMAP trial data Table 1.

**Table 1:** Application and adaptation to the PRALIMAP trial of the intervention dose determination framework

**Intervention Related Group (IRG) identification**	24 high schools * 3 strategies = 72 IRG: 36 IRG-A, 36 IRG-C
**Intervention periods identification**	2 intervention periods = intervention implemented during the grade 10 and 11 school years
**Identification and categorisation of the programme actors**	**Supervisors:** PRALIMAP monitors
	**Anchor personnel:** school professionals (administration staff, teachers, catering professionals, school nurses, …)
	**Targets:** high school students
**Indicator development**	**Non-programme-driven activities indicators:**
	* Developed for the 72 IRG
	* Concerned respectively the educational nutritional, screening and environmental activities performed independently of the PRALIMAP trial
	**Programme-driven activities indicators:**
	* Developed for the 36 IRG-A
	* Concerned the PRALIMAP activities planned by the frame of reference:
	- 12 IRG-Education: indicators investigated the delivery of lectures and collective works on nutrition and the participation in PRALIMAP meetings
	-12 IRG-Screening, indicators investigated the delivery of weight and height data and of the proposition to participate to adapted overweight care management and the participation of students in group educational sessions
	- 12 IRG-Environmental, indicators investigated the delivery of high school environment improvements (adapted food and physical activity availability) and participation in PRALIMAP parties
**Data collection**	**Data collected before the programme implementation:**
	* High schools nutritional environment (ex: water drinking fountain, proposed physical activities …) : nutritional surveys participated in by school staff
	* Nutritional behaviours : adolescent self-administered questionnaires and anthropometric measures
	**Data collected during implementation:**
	* Activities delivery data: activity reports, pupil satisfaction surveys (care management, PRALIMAP meeting…)
	* Appreciation of PRALIMAP trial : self administered questionnaire
	* Evolution of the offer of school catering and physical activity free equipment and the nutritional environment close by the high school: nutritional surveys participated in by school staff
	**Data collectedat the end of the programme:**
	* Activities delivery, school staff and teenagers’ participation and favouring and limiting factors :
	- focus group of staff responsible for interventional strategies (high school professionals, head teachers)
	- individual semi-structured interview of the PRALIMAP monitors
	- focus group of health professionals intervening with overweight and obese adolescents in high school screening
	- nutritional survey of high school professionals and students
**Data analysis and evaluation of indicators**	**Indicator report sheets are elaborated for every IRG including:**
	* Quantitative indicators expressed in the form of mean or percentage (eg : pupils' activity participation rate)
	* Qualitative (literal) indicators (eg : ranges of food proposed in the lunches, delivery or not of activity)
	**The number of indicator report sheets varied from 3 to 6 according to the high school assigned strategies** (Table 3) :
	*IRG–Education : 1 indicator report sheet of non-programme-driven activities + 1 indicator report sheet of programme-driven activities
	*IRG–Education control : 1 indicator report sheet of non-programme-driven activities
	*IRG– Screening : 1 indicator report sheet of non-programme-driven activities + 1 indicator report sheet of programme-driven activities
	*IRG–Screening control : 1 indicator report sheet of non-programme-driven activities
	*IRG–Environment : 1 indicator report sheet of non-programme-driven activities + 1 indicator report sheet of programme-driven activities
	*IRG–Environment control : 1 indicator report sheet of non-programme-driven activities
**Score assignment**	**Number of experts**:18 (3 groups of 6)
	**Type and specialty of experts:** researchers, field professionals or decision-makers, specialists in diet, physical activity and\or evaluation, knowing or not the PRALIMAP trial, practicing or not in Lorraine Region
	**IRG assigned between the experts**: the IRG were fairly and anonymously distributed among the experts
	**Individual scoring aid**: IT (Excel®)
	**Scoring :** ranging from 0 to 20 for every period, domain and characteristic in each IRG **Threshold** defined for the standard deviation and/or the range: if a standard deviation was higher than 2.5 or a range higher than 6 was observed, the experts debated and proposed a new notation; discrepant scores were then preserved.
	**Taking into account between-group variability**: A fictitious high school was created and scored by the 3 groups
**Intervention dose calculation**	**Application of intervention dose formula** to assigned scores: **Dose = DQt x (mean (DQl, PQt, PQl)/20)**
	A group effect has been evidenced thanks to the fictitious high school and required score adjustment varying from 0.8 to 2.8 points.
	Eventually 216 doses (108 per period) were calculated (Table 3)**.**

## Results and discussion

### Results

The framework and tool utilisation includes the following stages: identification of IRG, identification of intervention periods, identification and categorisation of programme actors, construction of indicators, data collection, data analysis and valuation of indicators, scoring, intervention dose calculation, and finally interpretation of implementation.

#### Intervention Related Groups (IRG) identification

The Intervention Related Groups (IRG) must be precisely identified by the investigator from the programme's inception. They represent the possible combinations of settings (for example schools, hospitals, cities, districts) and interventions (for example education, care, prevention) as defined in the programme. A setting intended to benefit from a particular intervention is referred to as IRG-Active (IRG-A), otherwise it is described as an IRG-Control (IRG-C) of the intervention concerned (Table 2).

**Table 2:** An example of intervention related group (IRG) identification

	**Setting 1**	**Setting 2**	**Setting 3**	**IRG**
Intervention 1	Yes	Yes	No	**3 IRG :**
				2 IRG-Active
				1 IRG-Control
Intervention 2	No	No	Yes	**3 IRG :**
				1 IRG-Active
				2 IRGs-Control
** IRG**	**2 IRG :**	**2 IRG:**	**2 IRG :**	**6 IRG :**
	1 IRG-Active	1 IRG-Active	1 IRG-Active	3 IRG-Active
	1 IRG-Control	1 IRG-Control	1 IRG-Control	3 IRG-Control

##### *PRALIMAP*

24 high schools (settings) were selected and three strategies (interventions) were evaluated, to give 72 IRG, among which 36 were IRG-A and 36 IRG-C.

#### Intervention periods identification

When a programme is implemented, it is important to divide it up (particularly if it is long) into manageable periods in order to reduce the effects of phenomena affecting those involved, such as tiredness, variations in the learning process, and changes in personnel.

##### *PRALIMAP*

Each adolescent benefited of interventions over two consecutive school years (grades 10 and 11) corresponding to two periods.

#### Identification and categorisation of the programme actors

Depending on the programmes, three categories of relevant people (supervisors, anchor personnel, targets) may or may not be present. The programme investigators comprehensively oversee the implementation but are not IRG-A supervisors and must not be so defined.

Anchor personnel receive training/information from supervisors, and then implement the intervention with the targets. They are often numerous, IRG-specific and occupy various posts and hierarchical positions. Information about events at anchor level is particularly important because that is where potential deviations from a programme's frame of reference originate: deviations such as not performing or only partially performing planned activities, and introducing unplanned activities.

Targets benefit from intervention and are the subjects of outcome measures.

##### *PRALIMAP*

The supervisors were the PRALIMAP monitors, the anchors were the high school professionals (administration staff, teachers, catering professionals, school nurses …) and the targets were the high school students.

#### Indicators development

Two types of indicator are required: specific indicators related to programme-driven activities, and general indicators related to non-programme-driven activities. The latter may lead to over- or under-estimation of programme-driven activities due to synergy or antagonism, respectively.

Programme-driven activities indicators were established for IRG-A, for every period and each of the 16 evaluation objects (Figure 1). Non-programme-driven activities indicators were developed for every IRG (including IRG-C, if any), every period, and every evaluation object.

##### *PRALIMAP*

Non-programme-driven activities indicators were developed for the 72 IRG and concerned the educational nutritional, screening and environmental activities performed independently of the PRALIMAP trial. Two examples of this type of activities can be given : eco-citizenship actions around nutrition took place in some of schools in the frame of the ‘Agenda 21’ plan ; actions (Sport, Wellness, first aid, breakfast, fruit…) has been implemented by some school staffs during local initiatives such as a ‘health week’.

Programme-driven activities indicators were established for the 36 IRG-A and concerned the planned PRALIMAP activities. Twelve IRG education indicators investigated the delivery of lectures and collective work on nutrition and participation in PRALIMAP parties. Twelve IRG screening indicators investigated the collection of weight and height data and information about intention to participate in adapted care management and the participation of students in group educational sessions. Twelve IRG environment indicators investigated improvements at high schools (changes in diet and physical activity available) and participation in PRALIMAP parties.

#### Data collection

Data collection relied on regular activity reports and on quantitative and qualitative investigations.

Activity reports permit monitoring of the quantity of intervention delivered and of participation in activities. They must be regularly completed by supervisors and providers.

The quantitative investigation of large target populations generally involves self-administered questionnaires, ideally completed at the same time as outcome measurement. It retrospectively assesses what has been done between two outcomes measurement points.

Qualitative investigation allows for measurement of delivery and participation and elucidates the interpretations and points of view of those involved. Collection methods are generally observation, collective interview (such as focus groups) and individual interview [3,18,19]

Both types of investigation complement one another and involve collection of information from the various people involved for every IRG and every period of intervention.

Data can be collected at various points:

before programme implementation to provide information about the initial context

during implementation, at the end of every period, to compare (in a concomitant or retrospective way) the performed activities to planned ones and to identify performed but not planned activities

at the end of the programme to assess general response and satisfaction.

The programme actors involved are the objects and the sources of information. For example, targets may report on their own participation and that of anchors.

##### *PRALIMAP*

Before the programme implementation, nutritional environmental data were collected at high schools via surveys of the staff. During implementation, delivery data were included in activity reports. Student satisfaction with the programme was measured using a self-administered questionnaire completed at the same time as outcome measurement and surveys of satisfaction with specific activities (care management, PRALIMAP party). Information about changes in school catering and physical activity supply, availability of free equipment, and the nutritional environment in the neighbourhood of the high school was assessed with a survey among the high school professionals. At the end of the programme, data on activity delivery, and on participation by school staff and students were collected by focus groups of staff responsible for interventional strategies (high school professionals, head teachers), and by individual semi-structured interview of PRALIMAP monitors.

#### Data analysis and valuation indicators

**Figure 2:**
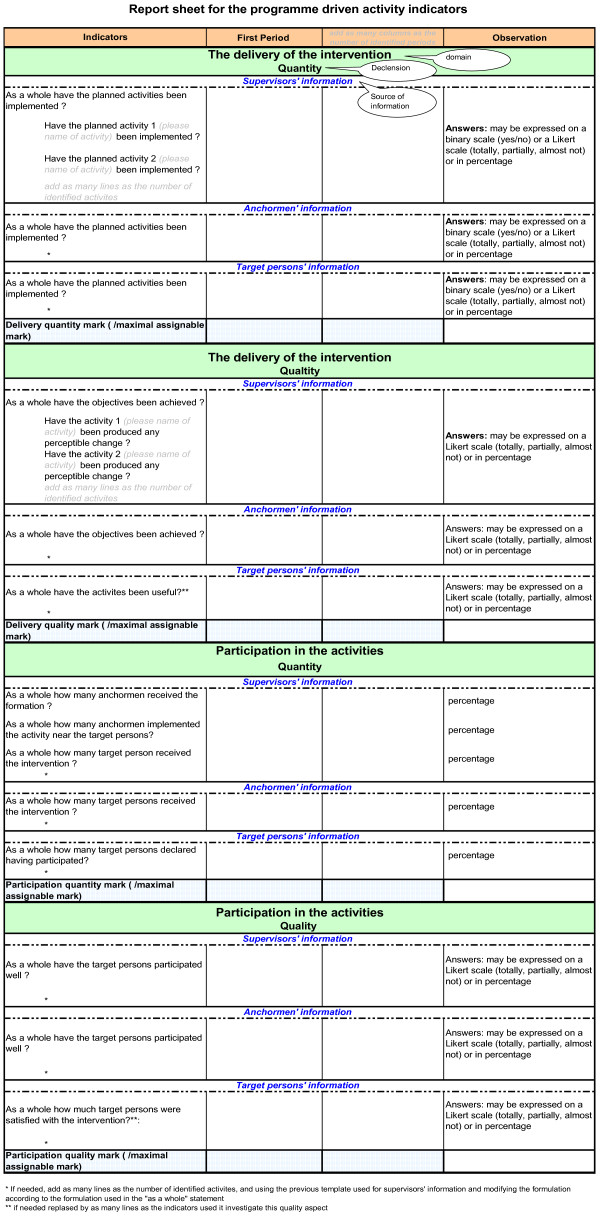
Template of indicators report sheet.

Data analysis allowed for valuation of the indicators developed. To facilitate the later expertise work, the valued indicators are bracketed within indicator report sheets. For every IRG, one or two indicator report sheets were elaborated, one covering non-programme-driven activities indicators and the other the programme-driven activities indicators (if IRG-A). On every indicator report sheet (Figure 2), indicators were presented by domain, declension, and programmes actors concerned as object and source of information, for each period of intervention.

##### *PRALIMAP*

Indicator report sheets were developed for every IRG and included quantitative indicators expressed in mean or percentage (eg: pupils' activity participation rate), qualitative (literal) indicators (eg: ranges of food proposed for lunches, delivery or not of activity). The number of indicator report sheets varied from three to six according to the high school assigned strategies (Table 3) totalling 72 indicator report sheets of non-programme-driven activities indicators and 36 of programme-driven activities indicators (IRG-A).

**Table 3:** Number and type of IRG and number of indicator report sheets and scores according to the high school and its assigned PRALIMAP strategies

**N° school**	**Strategy**			**Indicator report sheets**		
	**Education**	**Screening**	**Environment**	**Non-programme-driven activities**	**Programme-driven activities**	**Score total / school**
* 1*	IRG-C	IRG-C	IRG-C	3	0	**3**
* 2*	***IRG-A***	***IRG-A***	IRG-C	3	2	**5**
* 3*	***IRG-A***	***IRG-A***	***IRG-A***	3	3	**6**
* 4*	***IRG-A***	IRG-C	***IRG-A***	3	2	**5**
* 5*	***IRG-A***	IRG-C	IRG-C	3	1	**4**
* 6*	***IRG-A***	***IRG-A***	***IRG-A***	3	3	**6**
* 7*	***IRG-A***	***IRG-A***	IRG-C	3	2	**5**
* 8*	IRG-C	***IRG-A***	IRG-C	3	1	**4**
* 9*	***IRG-A***	***IRG-A***	***IRG-A***	3	3	**6**
* 10*	IRG-C	***IRG-A***	***IRG-A***	3	2	**5**
* 11*	***IRG-A***	IRG-C	IRG-C	3	1	**4**
* 12*	IRG-C	***IRG-A***	IRG-C	3	1	**4**
* 13*	IRG-C	IRG-C	IRG-C	3	0	**3**
* 14*	***IRG-A***	IRG-C	***IRG-A***	3	2	**5**
* 15*	IRG-C	***IRG-A***	***IRG-A***	3	2	**5**
* 16*	IRG-C	IRG-C	***IRG-A***	3	1	**4**
* 17*	***IRG-A***	***IRG-A***	IRG-C	3	2	**5**
* 18*	***IRG-A***	IRG-C	***IRG-A***	3	2	**5**
* 19*	IRG-C	IRG-C	***IRG-A***	3	1	**4**
* 20*	IRG-C	IRG-C	IRG-C	3	0	**3**
* 21*	IRG-C	IRG-C	***IRG-A***	3	1	**4**
* 22*	IRG-C	***IRG-A***	***IRG-A***	3	2	**5**
* 23*	***IRG-A***	IRG-C	IRG-C	3	1	**4**
* 24*	IRG-C	***IRG-A***	IRG-C	3	1	**4**
	***12 IRG Education***	***12 IRG*Screening**	***12 IRG*Environment**	**72**	**36**	**108**
	12 IRG –Control - Education	12 IRG –Control- Screening	12 IRG –Control- Environment			

#### Assignment of scores

We used the nominal group technique [20] to reach consensual scores. A score covers an IRG set of programme-driven or non-programme-driven activities indicators; it is assigned for every domain / declension and every period (Figure 2). It is impossible to establish from indicators (in particular those stemming from a qualitative investigation) an automatic scoring system. Collective expert techniques are the best methods in that context [21].

The collective expertise method is multidisciplinary, including decision-makers, professionals, researchers, and specialists in the topic of interest and\or the evaluation. The experts do not all have to be actively involved in the programme being assessed. Depending on the number of IRG concerned and available resources, one or several groups of at least six experts are constituted so as to obtain a variety of opinions. The notation sessions are managed by an independent moderator and take place in the following way:

anonymous presentation of the IRG characteristics to provide the experts with an overview of the environment in which the programme took place,

explication of the indicators and indicator report sheets,

determination of a theoretical range of scores,

IRG-blind scoring by the experts on an individual marking aid (IT or paper).

The mean, standard deviation and the range of scores assigned by the experts are calculated for every domain/declension and every period. If the standard deviation and/or the range exceed a previously agreed threshold, experts debate (under the moderator) in order to explain the deviations, and to look for a possible consensus. The debate leads to a second score. Mean scores are then preserved even in the absence of consensus [22].

When several groups of experts are constituted and in order to take into account the between-group variability, a fictitious IRG can be proposed to allow for a calibration.

Eventually, four IRG*period non-programme-driven activities scores and four IRG–A*period programme-driven activities scores are obtained for every period.

A wrap up debate needs to be performed with all the experts, in particular if several groups have been constituted. It allows for discussion of the relevance of scores, the difficulties encountered and the between-group variability, and preparation for the formal weighting of scores to be used for the dose calculations.

##### *PRALIMAP*

Three groups of six experts were constituted, comprising:

researchers, field professionals or decision-makers,

specialists in food, physical activity and/or evaluation,

people familiar or not with the PRALIMAP trial,

people practising or not in the Lorraine Region.

The experts assigned scores ranging from 0 to 20 for every period, domain and declension in each of the IRG, distributed fairly and anonymously among the experts. The scores were entered on computers, allowing for immediate display of results. If a standard deviation higher than 2.5 or a range higher than 6 was observed, the experts debated. A fictitious high school was created and scored by the three groups.

#### Intervention dose calculation

The four declensions are not independent but nested: participation is subject to delivery, and quality is subject to quantity. In practice, the impact of the quantity of activity delivery is likely to be decreased by the delivery quality as well as by the quantity and quality of participation. The delivery quantity score is therefore weighted by the mean of the delivery quality scores and of the participation quantity and quality score, the mean being divided by the common maximal assignable score.

**Thus Dose = DQt × (mean (DQl, PQt, PQl)/mas):**DQtdelivery quantity scoreDQldelivery quality scorePQtparticipation quantity scorePQlparticipation quality scoremascommon maximal assignable score

Two doses are calculated for every intervention period: one non-programme-driven activities dose for every IRG and one programme-driven activities dose for every IRG-A.

##### *PRALIMAP*

The formula was applied to scores assigned to each of the 72 IRG covering the 24 high schools. Overall, 216 doses (108 per period) were calculated: four for every IRG-A (a non-programme-driven activities dose and a programme-driven activities dose for each of the two periods) and two doses for every IRG-C (a non-programme-driven activities dose for each of the two periods).

A group effect revealed by the fictitious high school necessitated score adjustment varying from 0.8 to 2.8 points.

#### Implementation interpretation

The unit of analysis is the setting. For each setting, cluster characteristics (e.g. geographical zone, socioeconomic status) and target population characteristics (e.g. sex ratio, mean age, total number of professionals) were collected. Doses are expressed as means, medians, and distribution parameters. Doses calculated for an IRG are assigned to every target person belonging to it. The analysis allows for dose comparisons between IRG or IRG clusters as defined in the outcomes analysis plan.

##### *PRALIMAP*

The twelve mean doses (four for each of the three PRALIMAP strategies) obtained ranged from 5.2 (programme-driven activities dose second year screening) to 9.0 (non-programme-driven activities dose first year education) (Table 4).

**Table 4:** Global description of the intervention doses in the 24 high schools participating in the PRALIMAP trial

			**N**	**mean**	**standard deviation**	**median**	**Q1**	**Q3**	**min**	**max**
Education	NPDA*	year 1	24	9.0	3.5	8.3	6.6	12.5	3.4	13.9
		year 2	24	7.7	2.8	7.8	5.5	10.0	2.5	13.2
	PDA**	**year 1**	**12**	**8.2**	**1.9**	**7.8**	**7.3**	**9.3**	**4.7**	**11.8**
		year 2	12	6.3	3.5	7.5	2.7	8.5	0.5	10.9
Screening	NPDA	year 1	24	5.2	3.7	5.0	2.3	8.4	0.0	11.9
		year 2	24	5.0	3.4	5.4	1.8	7.9	0.0	10.1
	PDA	year 1	12	6.3	3.5	6.6	3.1	9.3	1.2	10.8
		year 2	12	3.6	2.7	3.0	1.9	4.8	0.5	8.8
Environment	NPDA	year 1	24	6.4	2.3	7.0	5.8	7.8	1.8	9.4
		year 2	24	5.7	2.0	6.1	4.6	7.3	1.2	9.6
	PDA	year 1	12	7.8	1.8	7.6	6.8	8.4	5.2	12.5
		year 2	12	8.2	3.1	8.6	6.9	9.7	0.3	12.1

* NPDA : Non-programme-driven activities.

** PDA : Programme-driven activities.

Variability of delivery from one high school to the other was evidenced for all the strategies; nutritional educational activities were performed in all the high schools allocated or not to the education strategy. A few active high schools performed practically no activity, in particular for the screening strategy.

The mean doses were low. The programme-driven activities mean dose of IRG-education for the first year was 8.2, while the mean doses of four constituent declensions / characteristics varied from 11.4 for the participation quality to 13.6 for the delivery quantity, with an IRG dose range from 6.5 for participation quantity to 15.8 for delivery quality (Table 5).

**Table 5:** IRG Education – year 1 detail of the mean assigned marks

		**N**	**mean**	**standard deviation**	**median**	**Q1**	**Q3**	**min**	**max**
Delivery	quantity	12	13.6	1.5	14.2	12.4	14.8	10.5	15.7
Delivery	quality	12	12.2	2.6	12.1	10.6	14.5	8.2	15.8
Participation	quantity	12	12.2	2.4	12.5	11.2	13.6	6.5	15.2
Participation	quality	12	11.4	2	11.2	9.8	12.8	8.7	14.5

The mean dose was higher in the first year than the second, with the exception of the programme-driven activities dose of the environment strategy. The median was lower than the mean except for environment. Control high schools had non-null intervention doses that were weak for the screening strategy (2.7 year 1), and higher for environment (5.6) and education (6.3). They benefited from interventions not planned by the programme. High schools that benefited from a strategy had doses significantly higher than their controls whatever the year. The general environment dose was significantly higher in IRG-A education than in IRG-C-education, and the educational non-programme-driven activities dose in the second year was significantly higher in IRG-A environment than IRG-C environment (Table 6).

**Table 6:** Mean doses obtained for each of the three PRALIMAP strategies

**Dose**		**EDUCATION**				**SCREENING**				**ENVIRONNEMENT**			
		**Control**		**Active**		**Control**		**Active**		**Control**		**Active**	
		**NPDA**	**PDA**	**NPDA**	**PDA**	**NPDA**	**PDA**	**NPDA**	**PDA**	**NPDA**	**PDA**	**NPDA**	**PDA**
Education	year 1	**6,3**		**11,7**	8,2	9,2	8,8	8,9	7,6	8,2	7,7	9,9	8,7
	year 2	**6,0**		**9,5**	6,3	8,0	6,2	7,5	6,5	**6,8**	5,5	**8,6**	7,2
Screening	year 1	6,1	6,4	4,4	6,2	**2,7**		**7,7**	6,3	5,5	6,3	4,9	6,3
	year 2	5,2	3,7	4,7	3,4	**2,5**		**7,4**	3,6	5,2	3,4	4,8	3,8
Environment	year 1	**5,2**	8,0	**7,6**	7,6	6,4	8,2	6,4	7,4	**5,6**		**7,2**	7,8
	year 2	**4,9**	8,9	**6,6**	7,5	5,7	8,3	5,7	8,0	**4,9**		**6,6**	8,2

***Bold face values****: statistically significant difference of the received dose between the control and the strategy groups*.

**Figure 3:**
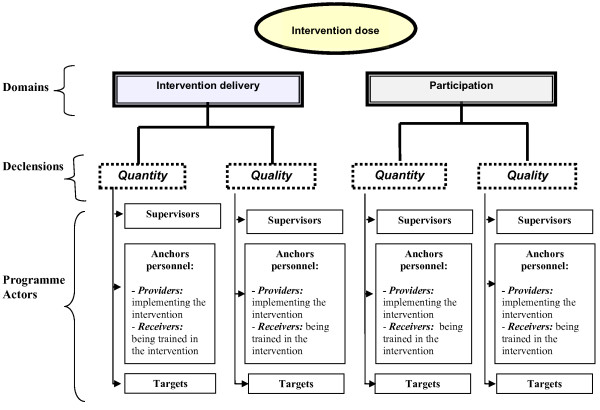
PRALIMAP educational and environmental intervention dose received according to the assigned strategies.

A significant negative interaction between the education and environment strategies emerged (Figure 3). When education and environment were implemented in combination, the doses of both were lower than expected in an additive model. The screening strategy was implemented independently of the other strategies (absence of interaction).

A multivariate analysis taking into account cluster characteristics (implementation waves, high school education type and geographical zone) and individual characteristics (gender, age, social and occupational status, BMI) did not modify the results.

### Discussion

A framework and a tool allowing for calculation of an implementation dose of programme- or non-programme-driven activities during health promotion programmes were elaborated, investigated and validated in a cluster randomized trial. An approach led by the theory necessitated specification of certain concepts (dose, delivery, participation, quantity, quality, programme actors, information sources), definition of new constructs (IRG, period, programme-driven activities, non-programme-driven activities) and development of information synthesis techniques (indicator report sheets by IRG, collective expertise, practical details of intervention dose calculation). Application in PRALIMAP confirmed the feasibility of the approach, demonstrated important implementation variability between IRG and over time, and showed that intervention doses can be obtained and used in future ‘in treatment’ analysis.

The importance of process in health programmes and trials has been increasingly recognised in recent decades and has been the subject of three important reviews [1-3]. In 1998, Dane and Schneider, reviewed 162 primary and secondary prevention studies [2]. They emphasised that failure to consider integrity data, particularly regarding adherence, can compromise the internal validity of prevention studies. In 2003, Dusenbury et al. [1], analyzed drug addiction prevention studies performed over a 25-year period. They revealed that poor implementation may reduce a programme's effectiveness and that strong methodologies to measure and analyze implementation should be developed. In 2008, Durlak et al. [3] reviewed more than 500 articles (the majority of which were already synthesised in five meta-analyses) and clearly showed that implementation level affects the outcomes of health promotion and prevention programmes. They contributed to the description of factors that influence implementation, and recommended implementation data collection, which they consider an essential feature of programme evaluation.

These three reviews showed that the terminology is not yet consolidated, probably hindering the dissemination of data from implementation studies. We used a pragmatic and general classification that covered the concepts used in the reviews (Table 7). They sometimes proposed components not of the same nature, for example exposure and programme differentiation. Indeed, programme differentiation is a peculiar characteristic that can influence implementation but does not depend on programme actors, whereas exposure represents the amount of the programme delivered. It is not always easy to distinguish, in papers, what is exactly meant by adherence, dose or quality. Our classification allows for a hierarchical organization of four components and thus for the calculation of what we call the ‘intervention dose’. These components are obtained by simply answering four questions: how much did providers do? Did providers do well? Did targets participate? And did targets participate well?

**Table 7:** Correspondence between the concepts used in this paper and three reviews

**Reviews**		**Dane and Schneider [**[2]**]**	**Dusenbury et al. [**[1]**]**	**Durlak et al. [**[3]**]**
**Legrand et al.**				
**Intervention dose**		**Integrity or Fidelity including 5 components :** exposure, adherence, quality of delivery, programme differentiation, participant responsiveness	**Fidelity including 5 components:** adherence, dose, quality of delivery, programme differentiation, participant responsiveness	**Including 8 coas mponents:** Fidelity, Dosage, Quality, Participant responsiveness, Programme differentiation, Contamination, Programme reach, Programme modification
**Delivery**	**Quantity**	Exposure	Dose	Dosage
				Fidelity (a k a : adherence or compliance or integrity, or faithful replication)
		Adherence	Adherence	
	**Quality**	Quality of delivery, Adherence	Quality of delivery, Adherence	Quality
**Participation**	**Quantity**	Participant responsiveness	Participant responsiveness	Programme reach
	**Quality**	Participant responsiveness	Participant responsiveness	Participant responsiveness
**Participants/ sources of information**	**Supervisors**	Supervisors,		
		Developers		
		Facilitators		
	**Anchors personnel (providers / receivers)**	Implementers (receivers) or providers	Providers	Providers
	**Targets**	Participants		
**IRG**		/	/	/
**Indicators**	**Non-programme- driven activities**	/	/	/
	**Programme-driven activities**	/	/	/

Like Dane and Schneider [2], we put the emphasis on clearly identifying, during the indicator construction process, the information sources and the various personnel involved in the programme – each of whom might be a source of information on the others. For example, in a school programme, students may assess the teachers’ participation and vice versa. That is why we suggest precisely identifying the people associated with each of the four components (Figure 1).

As underlined by Durlak et al. [3], no study has reported 100% implementation by providers. The implementation level depends on supervisors or providers, and varies from 20 to 40% depending on the setting. A supervisor or a provider operating in several programme settings can even behave differently in each. So it seems necessary to take into account the setting- and intervention-specific implementation level; hence we elaborated the new concept of IRG. In PRALIMAP, the variety, the number of supervisors and providers and the potential substitution of individual, from one school year to the next brought to light the importance of taking into account the period and the IRG. For the evaluation of an effectiveness trial, this notion is crucial to understanding of the relation between the implementation and the outcomes. It is just as important in health programmes not in the context of a trial in order to take account of variability and weaken the dilution effect induced by heterogeneity of settings.

Most studies consider only those activities directly driven by the programme. Durlak suggests considering the contamination aspect (treatment contamination, usual care, alternative services) in the level of implementation assessment, particularly when a control comparative group is used. We stress that implementation in a specific programme may be influenced by other concomitant programmes such as national media campaigns, local programmes or personal initiatives by those involved in the programme under consideration. Therefore, we distinguished between programme-driven and non-programme-driven activities relevant to the intervention under investigation.

PRALIMAP showed not only the importance of this distinction, particularly when estimating the effect of the intervention, but also the difficulty of distinguishing whether an activity (for example the delivery of a nutrition course within the curriculum) is performed in the programme's frame of reference. So, in high schools active for a given strategy, the non-programme-driven activities scores were higher than in control high schools when we could have expected them to be equal or even lower.

It is essential to have in mind the indicators from the programme inception to be sure to eventually have indicators for every domain, every declension and every person involved; the quality and the sufficiency of the data collection depend on that. So in PRALIMAP we were not able to collect data on participation in non-programme-driven screening activities.

Collective expertise appeared to be the most appropriate method [20,22,23] with which to facilitate dose calculation. In PRALIMAP, the experts underlined the importance of the first indicator sheet, which acts as a scoring reference. We observed between-group variability in scores thanks to the fictitious high school. To minimise variability, we recommend limiting the number of expert groups and submitting to the experts (without their knowing) a first indicator sheet corresponding to a fictitious IRG, which allows for measurement of the group effect and, if necessary, adjustment of scores.

Application to PRALIMAP confirms our hypothesis of strong implementation variability between IRG, with deviation depending on period and intervention strategy. Awareness of this variability is necessary in order to estimate the influence of implementation on programme outcomes [3]. That will be performed in PRALIMAP by ‘on-treatment’ analysis [24], in which the calculated dose of an IRG will be assigned to each student of that IRG. It is thus about a dose calculated collectively and not individually. The variability of the calculated final IRG dose may depend on the weighting method. The method we used reflects at best the implementation level in the target population but tends to reduce the dose measure variability. The ‘in treatment’ analysis could allow for validation of the proposed weighting method.

## Conclusions

The implementation of complex public health and health promotion programmes is measurable thanks to the calculation of an intervention dose. The calculation is based on the construction of indicators developed from the conception of the programme and rigorous data collection on the processes with programme actors likely to induce variations in the implementation.

Independent collective expert input ensures the validity of the measure obtained.

The tool can be used in any programme evaluation. It could be particularly useful in comparative trials and in studies of the influence of implementation on programme outcomes. Further developments and researches are needed to ensures its utility and evaluate its transferability to other contexts.

## Abbreviations

DQt: Delivery quantity score; DQl: Delivery quality score; IRG: Intervention related group; IRG-A: Intervention related group – active; IRG-C: Intervention related group – control; IT: Information technology; NPDA: Non-programme-driven activities; PDA: Programme-driven activities; PRALIMAP: Promotion de l'ALImentation et de l'Activité Physique; PQt: Participation quantity score; PQl: Participation quality score; mas: Common maximal assignable score.

## Competing interests

The authors declare that they have no competing interests.

## Authors' contributions

SB is the principal investigator of the PRALIMAP trial. JFC is the co-investigator. All authors are process evaluation managers. KL and EB constructed indicators, participated in data collection and evaluated indicators. FE prepared the information technology aid. EB, KL and SB moderated the three PRALIMAP notation sessions. CL, FE, JFC and NT were scorers. EB, SB and FE are statistical managers. EL is logistic head managers and EA is high school professional head managers. KL and SB. drafted the manuscript. All authors have read and approved the final manuscript. SB is the paper's guarantor.

## Pre-publication history

The pre-publication history for this paper can be accessed here:

http://www.biomedcentral.com/1471-2288/12/146/prepub

## Supplementary Material

Additional file 1 **Box.**The PRALIMAP trial. Click here for file
