# Supplementary material for: Intervention dose estimation in health promotion programmes: a framework and a tool. Application to the diet and physical activity promotion PRALIMAP trial
Source: BMC Med Res Methodol. 2012 Sep 19;12:146. doi: 10.1186/1471-2288-12-146 (PMC3561200; doi:10.1186/1471-2288-12-146)
Supplement: Additional file 1 — Box.The PRALIMAP trial. [file 1471-2288-12-146-S1.doc]

**Box:** **The PRALIMAP trial**

***Objective***

PRALIMAP (PRomotion de l’ALIMentation et de l’Activité Physique) was a 2x2x2 factorial cluster randomised trial to evaluate the effectiveness of three health promotion strategies – educational, screening and environmental – applied singly or in combination to promote healthy dietary habits and physical activity among adolescents in high school over a two-year intervention period. The methods used in PRALIMAP are described in detail elsewhere [6] and briefly reported below. PRALIMAP began after the tool to estimate intervention dose was designed.

***Participants***

The PRALIMAP trial group randomly selected 24 state-run high schools. Adolescents entering the selected high schools in grade 10 in 2006 or 07 (according to the school) and in grade 11 in 2007 or 08 benefited from interventions; they were included in the trial outcomes evaluation.

***Design of PRALIMAP***

The interventional strategies were organized over two academic years between 2006 and 2009, and follow-up consisted of three assessment visits: at the start of grade 10 (T0), grade 11 (T1) and grade 12 (T2).

Due to the 2x2x2 factorial randomisation of the 24 high schools, three each were assigned to receive the educational and environmental strategies, the educational and screening strategies, the screening and environmental strategies, the educational strategy alone, the environmental strategy alone, the screening strategy alone, all three strategies, or no intervention.

***PRALIMAP Interventions***

Three prevention strategies were used: educational strategy, screening strategy and environmental strategy. “Educational strategy” involved developing personal skills to adopt healthy behaviours in the context of nutrition (diet and physical activity) according to current guidelines. The Education Strategy included three types of activity. First, dietary and physical activity lectures, occupying five hours in grade 10 and six in grade 11, were provided by high school teachers of life sciences and/or physical education. Second, adolescents performed group work in which they had to exchange, find and present to their classmates their own answers to problems related to eating habits, physical activity and the environment. These collaborative projects, conducted in both grades 10 and 11, were supervised by teachers and PRALIMAP monitors. Third, two PRALIMAP parties were organized, one at the end of each school year. During these parties, several activities were proposed (e.g., fun physical activities, games, tests, conferences, food and drink tasting) in order to reinforce what they had learned about healthy food choices and to be physically active in a convivial atmosphere. High school professionals and students were invited to organize and to participate in the event.

“Screening strategy” meant measuring, detecting overweight/obesity and eating disorders, and proposing, if necessary, adapted care management.

The Screening Strategy consisted of detecting overweight / obesity and/or eating disorders in students by the school nurse and proposing, if necessary, an adapted care management of seven 1.5-hour group educational sessions conducted inside or outside high schools by a nutrition network. Nutrition networks are associations of professionals specialized in overweight and obesity management (physicians, dieticians, psychologists and sports educators), and located in the neighborhood of the high school. These sessions covered food and physical activities, and changes in nutritional habits, and aimed to inform adolescents and answer their questions about nutrition and weight.

“Environmental strategy” meant developing favourable and supportive environments for healthy behaviours targeting the catering service of the school and relevant school policy.

The Environment Strategy consisted of increasing the availability of fruits, vegetables, bread and dairy products, water and physical activity. Activities such as presenting a menu with the food group colors or producing posters on available physical activities were also implemented. Moreover, a project committee including the school headmaster, high school professionals and the PRALIMAP monitors was charged with implementing features and activities to improve the nutritional environment. The final activity of this strategy was the PRALIMAP party as described for the education strategy, except that students did not participate in the organization.

The three strategies were implemented in high schools according to standard operating procedures. The educational and environmental strategies were managed by PRALIMAP monitors, trained health education professionals external to the schools and specifically recruited for the trial. The monitors clarified objectives to be reached, proposed and initiated activities and accompanied and supported high school professionals. The screening strategy was managed by public health professionals of the University of Lorraine, high school nurses, practitioners and an external nutrition health network.
